# Acute Effects of a Whole Body Vibration Session on the Vibration Perception Threshold in Patients with Type 2 Diabetes Mellitus

**DOI:** 10.3390/ijerph17124356

**Published:** 2020-06-18

**Authors:** Francisco Javier Dominguez-Muñoz, Miguel Angel Hernandez-Mocholi, Santos Villafaina, Miguel Angel García-Gordillo, Daniel Collado-Mateo, Narcis Gusi, Jose Carmelo Adsuar

**Affiliations:** 1 Physical Activity and Quality of Life Research Group (AFYCAV), Faculty of Sport Science, University of Extremadura, 10003 Cáceres, Spain; fjdominguez@unex.es (F.J.D.-M.); mhmocholi@unex.es (M.A.H.-M.); svillafaina@unex.es (S.V.); ngusi@unex.es (N.G.); 2 Facultad de Administración y Negocios, Universidad Autónoma de Chile, sede Talca 3467987, Chile; 3 Centre for Sport Studies, Rey Juan Carlos University, Fuenlabrada, 28943 Madrid, Spain; danicolladom@gmail.com; 4 Health Economy Motricity and Education (HEME), Faculty of Sport Science, University of Extremadura, 10003 Cáceres, Spain; jadssal@unex.es

**Keywords:** acute effects, diabetes mellitus, vibration perception threshold, whole body vibration

## Abstract

Background: Type 2 Diabetes Mellitus (T2DM) is a chronic disease that affects millions of people, and according to the International Diabetes Federation, 46.5% of people have undiagnosed diabetes. One of the most common complications of diabetes mellitus is loss of peripheral sensation. Whole Body Vibration (WBV) is a therapy, and it would be interesting to know if it can be considered as a training method to improve the Vibration Perception Threshold (VPT). The aim of the study is to verify whether there are really acute effects on the VPT after a WBV training session in people with T2DM. Methods: Ninety people with T2DM (56 men and 34 women) were randomly allocated to two groups: the WBV group and the placebo group. The ninety subjects went through a VPT training test before receiving the assigned intervention, and they performed the VPT test using the Vibratron II device. Results: After one session of WBV, an increase of the VPT in the WBV group was found, with respect to the placebo group. Conclusions: Vibration perception threshold is increased after a WBV training session in people with T2DM, compared to a placebo group.

## 1. Introduction

Type 2 Diabetes Mellitus (T2DM) is a chronic disease that affects millions of people. It is characterized as a state of hyperglycemia in fasting or postprandial states [1,2].

According to epidemiological studies, 415 million people in the world are affected by T2DM [3], but this could be an underestimation, since, according to the International Diabetes Federation, there are 46.5% of people with T2DM still undiagnosed. In Spain, one of the main causes of this large number of people with T2DM is the increasingly aged demographic structure, since this is considered a risk factor for the disease [4].

Several complications are frequently associated with T2DM, such as peripheral neuropathy. This condition is defined as “peripheral, somatic, or autonomic nervous damage”, of several distinct clinical entities as diffuse neuropathies (symmetric distal sensorimotor polyneuropathy and autonomic neuropathy) and focal neuropathy (mononeuropathy, plexopathy, radiculopathy, and cranial neuropathy). T2DM is the main cause of peripheral neuropathy, which is primarily a sensorial neuropathy that starts in the distal part or the lower extremities [5]; 50% of the people affected by T2DM for a long time present this kind of Diabetic Peripheral Neuropathy (DPN) [6].

DPN is characterized by a progressive loss of sensitivity in the distal parts of the body, because of the damage to the small-diameter cutaneous nociceptive fibers [7]. In addition, the neuromotor fibers can suffer damage with the associated loss in muscular strength. For instance, it has been seen that people with T2DM have a 17% and 14% reduction in muscular strength in knee flexor and extensor muscles, respectively [8]. It is common that, as a consequence of these alterations in the peripheral somatic and motor systems, the proprioception and sensitivity, mainly in the ankle and feet were altered [9,10], and therefore also the balance, posture, and locomotion [11]. Therefore, it is important to perform treatments to improve the function of peripheral nerves, and, of course, to control the blood glucose [12].

Whole Body Vibration (WBV) is a physical activity modality which consists of the application of an oscillatory force, where energy is transferred from an actuator (the vibration device) to the human body [13]. This therapy has been used to improve physical conditions in different populations, particularly in people with neurological and/or musculoskeletal problems [14].

WBV has been used to enhance balance and muscle strength in T2DM patients [14,15], and there are at least two systematic reviews and one meta-analysis that summarize the results of the research into the effects of training T2DM people with WBV [16,17].

To our knowledge, no therapy has been found to reverse neuropathy. We have not found scientific studies showing short-term effects or acute effects of WBV training on the Vibration Perception Threshold (VPT) in people with T2DM.

However, four articles have been published that have found encouraging results regarding the capacity of WBV training in improving the VPT. These studies are about long-term effects on people with low back pain [18], and for people with T2DM [19]. Only the studies of Hernández-Mocholi et al. [20] and Schlee et al. [21] have delved into the acute effects of WBV on VPT in healthy young people, and no study shows the acute effects of a WBV session in people with T2DM.

The purpose of this study is to verify whether there are indeed acute effects on the VPT after a WBV training session in people with T2DM.

## 2. Materials and Methods

### 2.1. Study Design

A double-blind, randomized controlled trial was carried out (ISRCTN16866781). The study was approved by the University of Extremadura Bioethical Committee (44/2012).

### 2.2. Study Participants

**Figure 1:**
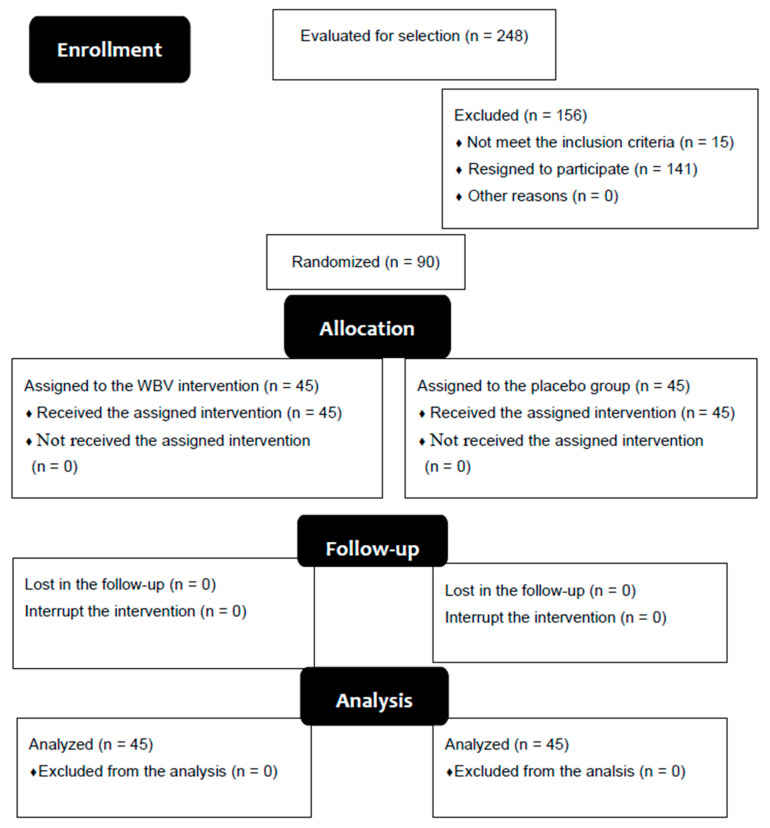
Flow diagram.

Ninety patients with T2DM met the inclusion criteria to participate in this study, and were randomly allocated into two groups: the WBV group (45 participants) and the placebo group (45 participants). There was no experimental death in the study (see Figure 1).

The inclusion criteria of the study were: (1) men and women with T2DM diagnosis between 40 and 85 years old; and (2) have accepted to participate in the study and signed the informed consent.

The exclusion criteria of the study were: (1) have contraindications to participate in high performance exercises such as retinopathy, musculoskeletal limitations, serious balance problems, or high thrombosis risk; (2) be under psychotropic or neurotoxic treatment; (3) be exposed to neurotoxins (industrial accidents, toxic residues); (4) be treated with radiation therapy; (5) non-diabetic neuropathic high risk (HIV AIDS, uremia, alcoholism); (6) have, or have had, a job with high exposure to mechanical WBV; and (7) have performed WBV exercises prior to intervention.

### 2.3. Procedure

The ninety participants went through a VPT training test before receiving the assigned intervention (WBV or placebo).

WBV group training: this group had a 12-min WBV exercise; the session parameters are shown in Table 1.

WBV was provided with a Galileo 900 (Novotec Medical GmbH, Pforzheim, Germany). This device makes fast oscillating movements around the sagittal. Once on the platform, the participants had to follow the following instructions: (1) stand on the platform on their feet at an equal and standardized distance from the middle axis (4 mm of vibration amplitude); (2) keep their eyes fixed forward with the back straight; (3) maintain a knee flexion at 45°; and (4) get on the platform without shoes.

Placebo group training: the placebo group were told that they were receiving an underneath vibration threshold that could not be detected. So, they got on the Galileo Fitness platform that was wired and connected to a screen, upon which the participant was able to see the vibration duration values, the frequency, and amplitude, but this really was a software control of loudspeakers put into the vibration platform. A similar placebo was previously used [20,22]. The trainer instructions given to the participants every time they performed the exercise were similar to those given to the WBV group.

### 2.4. Outcome

The VPT was the main study measure. It was evaluated before starting the vibration and immediately after each training session. VPT was assessed using the Vibratron II (Physitemp Instruments, Clifton, NJ, USA). The device produces vibration amplitudes from 0.005–200 microns, expressed as vibration units (0.005 microns = 0.1 vibration unit; 200 microns = 20.0 vibration units), with a higher vibration unit value indicating worse performance or greater sensory dysfunction.

The vibration units are the Vibraton II measure units. These vibration units are related to the movement amplitude measured in microns, and they follow the following formula:

A = x^2^/2 (where x is the vibration units [vu] and A is the amplitude in microns [μ]).

The “two-alternative forced-choice procedure” was the measure protocol, and the final VPT calculation used in this study. This protocol is recommended by the manufacturer, and it has been previously used in several studies [23,24].

### 2.5. Randomization

The randomization ratio was 1:1. A technician that did not belong to the members of the research team allocated the participants either to the experimental group (WBV) or to the placebo group, following a random selection algorithm.

### 2.6. Blinding

Neither the patients nor the investigation team members knew which persons were allocated to the placebo group or to the experimental group.

To protect the investigation team members’ blinding, the technicians in charge of the procedure were not members of the investigation team. During the statistical analysis, none of the investigators knew to which group had been allocated each of the study participants.

### 2.7. Statistical Analysis

Means and standard deviation were calculated for the entire sample. These were segregated by WBV group and placebo group.

The Kolmogorov–Smirnov test was used to check the data distribution. The following variables followed a parametric distribution: height and vibration perception threshold; the remaining variables did not follow a parametric distribution: age, weight, HbA1c, and years of diagnosis. In baseline, a student’s *t*-test was used to check the statistically significant differences, and a Mann–Whitney U test was used for the non-parametric variables.

Additionally, the analysis of covariance (ANCOVA) test was used to know whether or not age, height, sex, and VPT baseline had an effect on the outcome variable (VPT) after removing the variance for quantitative predictors (covariates). The SPSS statistical package (version 21.0; SPSS, Inc., Chicago, IL, USA) was used to analyze the data.

Cohen’s d was used to calculate effect size, and was classified as follows: values below 0.2 corresponded to a small effect size, values greater than 0.2 and less than 0.8 corresponded to medium effect size, and values greater than 0.8 corresponded to a large effect size [25].

## 3. Results

Table 2 shows the characteristics of the participants in the study. It can be seen that there were no statistically significant differences in the variables assessed between the WBV group and the placebo group at baseline.

Table 3 shows the acute effects of a WBV session on the VPT, with statistically significant differences in the VPT. After one session of WBV, the VPT increased in the WBV group, with respect to the placebo group. Age, VPT baseline, and height mediate in the WBV training effect on the VPT.

## 4. Discussion

The main finding of this study is that there are statistically significant acute effects, after one session of WBV, in the VPT in the WBV group, compared to the placebo group. To our best knowledge, this is the first study where the acute effects on VPT of a WBV session in patients with T2DM have been analyzed.

In line with the results obtained in this study on the increase of VPT after a session of WBV in this research, there are two previous studies where the acute effects of WBV on VPT have also been analyzed. In this regard, Hernández-Mocholi et al. [20] found increases in the VPT in the WBV group of up to 97%, and Schlee et al. [21] found an increase in the VPT of 55%, while this study found an increase of 7.64% in the VPT of the WBV group. These differences in percentage increases could be mainly due to three factors: (1) differences in the volume and intensity of the WBV session; (2) the type of target population in which the study is conducted; and (3) the age of the study participants.

With regard to the volume and intensity of the WBV session, the intervention sessions of the articles by Hernández-Mocholi et al. [20] and Schlee et al. [21] were of the same duration and intensity (4 min at 27 Hz); however, in this study a session of eight series of 30 s at a vibration frequency of 12.5 Hz was carried out. Regarding the type of sample, the studies by Hernandez-Mocholi et al. [20] and Schlee et al. [21] had samples of healthy young people, while in this study, the population is people with T2DM. Finally, with regard to the average age of the participants, in the studies by Hernandez-Mocholi et al. [19] and Schlee et al. [20], the ages were between 18 and 40 years and 25.3 years, respectively; however, in this study the average age is 65.6 years.

Another finding of this study is that the effect of WBV training on VPT is mediated by the different covariates, such as age, threshold baseline, and height of the population that comprises this study. Previous studies have linked age to VPT [26,27,28]. These studies corroborate that age is the major determinant of VPT. Loss of sensation may be due to the fact that Pacinian corpuscles deteriorate with age [29,30]. Another reason is that with age, the afferent spinal tracts that mediate vibratory stimuli progressively lose their ability to transmit information about the amplitude of the stimuli to the central nervous system [31].

With regard to height, there are numerous studies that corroborate that height influences the VPT [32,33,34], with height being considered a risk factor for having worse values in the VPT [32,33,34]. This may be due to the fact that height influences the speed of the motor nerves [35,36], as they are longer. For people with DM, the risk of diabetic polyneuropathy increases with the loss of nerve fibers and decreased sensation in more distant parts of the body [37,38,39]. Although there are differences in the VPT according to sex, in the ANCOVA analysis performed, it was not shown to mediate the effect of the WBV session on the VPT.

Taking into account the study results, it is possible that Pacinian corpuscles are acutely accommodated to the stimulus, progressively decreasing the rate of action potentials, due to a constant stimulus [40]. Regarding Pacinian corpuscle, the nerve terminal is surrounded by a many-layered capsule in an “onion skin” way. This structure acts as a mechanical filter which prevents slow displacements from deforming the nerve ending at the center of the corpuscle [41]. Pacinian corpuscles are present in the subcutaneous tissue of the foot and toe pads (4–6 mm below the skin surface) [42]. Interestingly, this corpuscle usually responds with one action potential for each sinusoid wave to vibration in a range of 150 Hz to 400 Hz. In this regard, Pacinian corpuscles will fire in response to indentations of 1 μm or less [43], conducting at about 45–65 m⋅s^−^^1^ [44]. Therefore, results are quite relevant, since WBV exercises could temporally inactivate these mechanoreceptors. This has crucial implications in populations with peripheral neuropathies, such as people with T2DM [6] after WBV exercises.

In this regard, one application that could be drawn from this study is that by worsening the capacity of the somatosensory system after receiving a WBV session, it could worsen balance and increase the risk of falls in people after training. However, this cannot be affirmed, since in the study by Schlee et al. [21], it was seen that although the threshold increased after the application of WBV, the balance improved. On the other hand, it would be of vital importance to know how long the acute effect of undergoing WBV on the VPT lasts, in order to determine the appropriate recommendations for a WBV training therapy. Some authors have hypothesized that the general adaptation syndrome theory [45] could be applicable to VPT. According to this theory, the body adapts to a stimulus, which initially causes a worsening of performance, and then adapts and has a subsequent improvement in performance to face a new stimulus in better conditions. Thus, further studies should investigate the duration of this acute effect on the VPT.

The current study has some limitations that should be taken into account. One of the limitations is that the appropriate dose–response to cause improvements in VPT in patients with T2DM has not been considered in this study. Only the acute effects of a single session of WBV have been studied. It would be appropriate to conduct a future study where acute effects can be seen during several sessions of WBV, and to better see the dose–response of WBV in a population with T2DM.

Additionally, one of the major limitations is that the mechanism by which the VPT could be improved through WBV training is not studied.

## 5. Conclusions

Vibration perception threshold is increased after a whole body vibration training session in people with type 2 diabetes mellitus, compared to a placebo group. This increase in vibration sensitivity threshold indicates greater sensory dysfunction.

## Figures and Tables

**Table 1:** Whole body vibration group training session parameters.

Intervention time (minutes)	12
Number of series	8
Time of each series (seconds)	30
Vibration frequency (Hertz)	12.5
Rest between series (seconds)	30

**Table 2:** Baseline characteristics of the study participants.

	All (*n* = 90)	WBV Group (*n* = 45)	Placebo Group (*n* = 45)	*p*
Age (years)	65.64 ± 8.65	65.00 ± 8.98	66.28 ± 8.36	0.510 *
Height (cm)	164.89 ± 10.01	163.91 ± 9.73	165.87 ± 10.28	0.357 **
Weight (kg)	80.63 ± 16.19	81.78 ± 18.03	79.47 ± 14.22	0.926 *
HbA1c (%)	6.78 ± 1.02	6.77 ± 1.15	6.79 ± 0.88	0.572 *
Years of Diagnosis	9.96 ± 8.83	9.53 ± 9.21	10.38 ± 8.52	0.352 *
VPT (vu)	5.66 ± 2.55	5.33 ± 2.26	5.98 ± 2.80	0.235 **

Data are expressed as mean and standard deviation; WBV: whole body vibration; cm: centimeters; kg: kilograms; HbA1c: glycosylated hemoglobin; VPT: vibration perception threshold; vu: vibration units. * The *p* values were calculated through a Mann–Whitney U test. ** The *p* values were calculated through a *t*-test for independent samples.

**Table 3:** Effects of a WBV session on the VPT in people with T2DM (*n* = 90).

	WBV Group (*n* = 45)		Placebo Group (*n* = 45)		Differences between Interventions [Mean (95% CI)]	*F*	*p* *	Effect Size
	Pre Mean (SD)	Post Mean (SD)	Pre Mean (SD)	Post Mean (SD)				
VPT (vu)	5.34 (2.27)	5.75 (2.26)	5.98 (2.80)	5.99 (2.84)	0.40 (From 0.003 to 0.801)	4.027	0.030	0.42
Age						4.792	0.031	
VPT Baseline						6.648	0.012	
Gender						2.048	0.156	
Height						4.254	0.042	

T2DM: Type 2 diabetes mellitus; WBV: whole body vibration; CI: confidence interval; VPT: vibration perception threshold; vu: vibration units; SD: standard deviation. * The *p* values were calculated through an analysis of covariance (ANCOVA) for repeated measurements, using the VPT baseline as a covariate. This covariate was significant, with a *p* of 0.006.
